# Beyond base excision repair: an evolving picture of mitochondrial DNA repair

**DOI:** 10.1042/BSR20211320

**Published:** 2021-10-14

**Authors:** Kathrin Allkanjari, Robert A. Baldock

**Affiliations:** 1 Formerly: Solent University Southampton, East Park Terrace, Southampton, SO14 0YN, UK; 2 School of Natural and Social Sciences, University of Gloucestershire, Francis Close Hall, Swindon Road, Cheltenham GL50 4AZ, UK

**Keywords:** DNA replication and recombination, DNA synthesis and repair, genome integrity, mtDNA

## Abstract

Mitochondria are highly specialised organelles required for key cellular processes including ATP production through cellular respiration and controlling cell death via apoptosis. Unlike other organelles, mitochondria contain their own DNA genome which encodes both protein and RNA required for cellular respiration. Each cell may contain hundreds to thousands of copies of the mitochondrial genome, which is essential for normal cellular function – deviation of mitochondrial DNA (mtDNA) copy number is associated with cellular ageing and disease. Furthermore, mtDNA lesions can arise from both endogenous or exogenous sources and must either be tolerated or corrected to preserve mitochondrial function. Importantly, replication of damaged mtDNA can lead to stalling and introduction of mutations or genetic loss, mitochondria have adapted mechanisms to repair damaged DNA. These mechanisms rely on nuclear-encoded DNA repair proteins that are translocated into the mitochondria.

Despite the presence of many known nuclear DNA repair proteins being found in the mitochondrial proteome, it remains to be established which DNA repair mechanisms are functional in mammalian mitochondria. Here, we summarise the existing and emerging research, alongside examining proteomic evidence, demonstrating that mtDNA damage can be repaired using Base Excision Repair (BER), Homologous Recombination (HR) and Microhomology-mediated End Joining (MMEJ). Critically, these repair mechanisms do not operate in isolation and evidence for interplay between pathways and repair associated with replication is discussed. Importantly, characterising non-canonical functions of key proteins and understanding the bespoke pathways used to tolerate, repair or bypass DNA damage will be fundamental in fully understanding the causes of mitochondrial genome mutations and mitochondrial dysfunction.

## Introduction

Mitochondria are highly specialised and dynamic organelles required for fundamental cellular processes including ATP generation via oxidative phosphorylation during cellular respiration and the control of programmed cell death by apoptosis (reviewed in [1]). Unlike other mammalian organelles, mitochondria contain their own 16.5 kb circular DNA genome (often referred to as mitochondrial DNA or mtDNA) comprising 37 genes which in turn encode 13 peptides required for the respiratory chain complexes (I–IV) and ATP synthase [2]. A further 22 transfer RNAs and 2 ribosomal RNAs enable protein synthesis of these proteins within the mitochondria [3]. Compartmentalised in the mitochondrial matrix, each cell is estimated to contain hundreds to thousands of copies of the mitochondrial genome dependent on the cell type and between eight and ten copies per mitochondrion [3]. This genetic material is clustered and organised into distinct nucleoid structures marked predominantly by association with mitochondrial transcription factor A (TFAM) and several other mtDNA-associated proteins [4,5]. Mitochondrial nucleoids are associated with the inner mitochondrial membrane and support mtDNA packaging, replication and mediate signalling (reviewed in [6]).

Critically, mtDNA loss or a decrease in the number of functional copies of the mitochondrial genome has been shown to cause multiple human pathologies including a group of heritable syndromes referred to as mitochondrial DNA depletion syndromes (MDDSs) which are often characterised by neurological features, hypotonia and gastrointestinal problems identifiable in infancy [7]. These syndromes and the underlying mechanism of disease, arise from defects not within the mitochondrial genome, but within nuclear genes that function to protect the genetic integrity of the mtDNA [8].

One of the best studied examples of nuclear-encoded proteins maintaining the integrity of the mitochondrial genome is polymerase γ (POLγ) which was originally thought to be the sole polymerase responsible for replication of the mitochondrial genome [9]. Mutations in the nuclear genes encoding subunits of POLγ, *POLG* and *POLG2*, that affect the proofreading capability of the polymerase have been shown to elevate misincorporation of bases and mutagenesis in the mitochondrial genome and accelerate cellular ageing [10–12]. As a result, cataloging variations in the POLG gene are of particular interest in tracking increased genomic instability of the mitochondrial genome and associated diseases (reviewed by [13]). In addition, altered mitochondrial genome copy number has also been observed in human cancers as demonstrated by analysing data from The Cancer Genome Atlas [14]. Though it remains to be established whether this contributes to the disease phenotype or occurs because of the abnormal cellular physiology. In either case, mitochondrial genome copy number and integrity of mtDNA must be carefully maintained to protect mitochondrial function.

Despite the approximately ten-fold elevated rate of mutation, mitochondrial function is arguably more resilient to individual genetic changes in single copies of the mitochondrial genome such as single-nucleotide polymorphisms (SNPs) and mutations due to the large copy number [15]. Unlike the diploid nuclear genome with only two copies of each gene, base changes in a small proportion of the total number of copies of the mitochondrial genome are unlikely to yield a negative impact or lead to disease due to the presence of hundreds to thousands of additional copies capable of compensating for the genetic change [3]. The presence of both normal and abnormal copies of the mitochondrial genome within a single cell is referred to as heteroplasmy and is estimated to only affect 1–2% of the total copy number [16]. In contrast, homoplasmy describes when all copies of the mitochondrial genome within a eukaryotic cell are identical, this term can be applied to both uniformly normal and abnormal mtDNA sequences assuming they are the sole variant present [16]. Critically, mitochondrial heteroplasmy has been linked to diseases such as cancer and can play a role in other conditions through the ‘threshold’ effect [17]. In the threshold model, heteroplasmy may not lead to disease phenotypes unless the ratio of normal to abnormal mtDNA shifts across a threshold at which point the disease phenotype becomes apparent [16,18]. The role of heteroplasmy in disease, however, is likely more complex with variable levels of heteroplasmy having been identified in different human tissues alongside associations with cellular ageing, neurodegenerative diseases and cancers [19–21]. Mechanistically, the proteins that govern and maintain integrity of the mitochondrial genome either through DNA replication or repair are of particular interest for understanding the aetiology of mitochondrial dysfunction and disease.

## Mitochondrial localisation of nuclear-encoded DNA repair genes

Characterising the impact of proteins, encoded by nuclear genes, on mitochondrial function and protection of mtDNA is largely challenging as many of these genes do not appear to contain a clear consensus mitochondrial localisation signal peptide [22]. Many proteins are found in multiple cellular compartments, which in the case of mitochondria often involves a mitochondrial localisation signal encoded within the peptide sequence of the protein, such as alternative splicing of the DNA glycosylase, 8-Oxoguanine DNA Glycosylase (OGG1) [23,24]. Alternative splicing as well as alternative transcript and translation start sites also give rise to multiple transcript variants which can dictate the localisation of a protein via the inclusion/exclusion of mitochondrial localisation signals respectively [24]. Other characterised examples include DNA ligase III, which functions in both nuclear and mitochondrial compartments and contains an upstream, in-frame start codon that leads to the inclusion of a mitochondrial targeting peptide when used to initiate translation [25]. In contrast, alternative splicing and use of alternative transcription initiation sites of the *UNG* gene, encoding the uracil DNA glycosylase, permits the inclusion of the targeting sequence [26,27]. However, many more proteins that are targeted to the mitochondria have yet to have a defined consensus targeting sequence attributed to them or may be trafficked with other proteins containing localisation signals.

**Figure 1:**
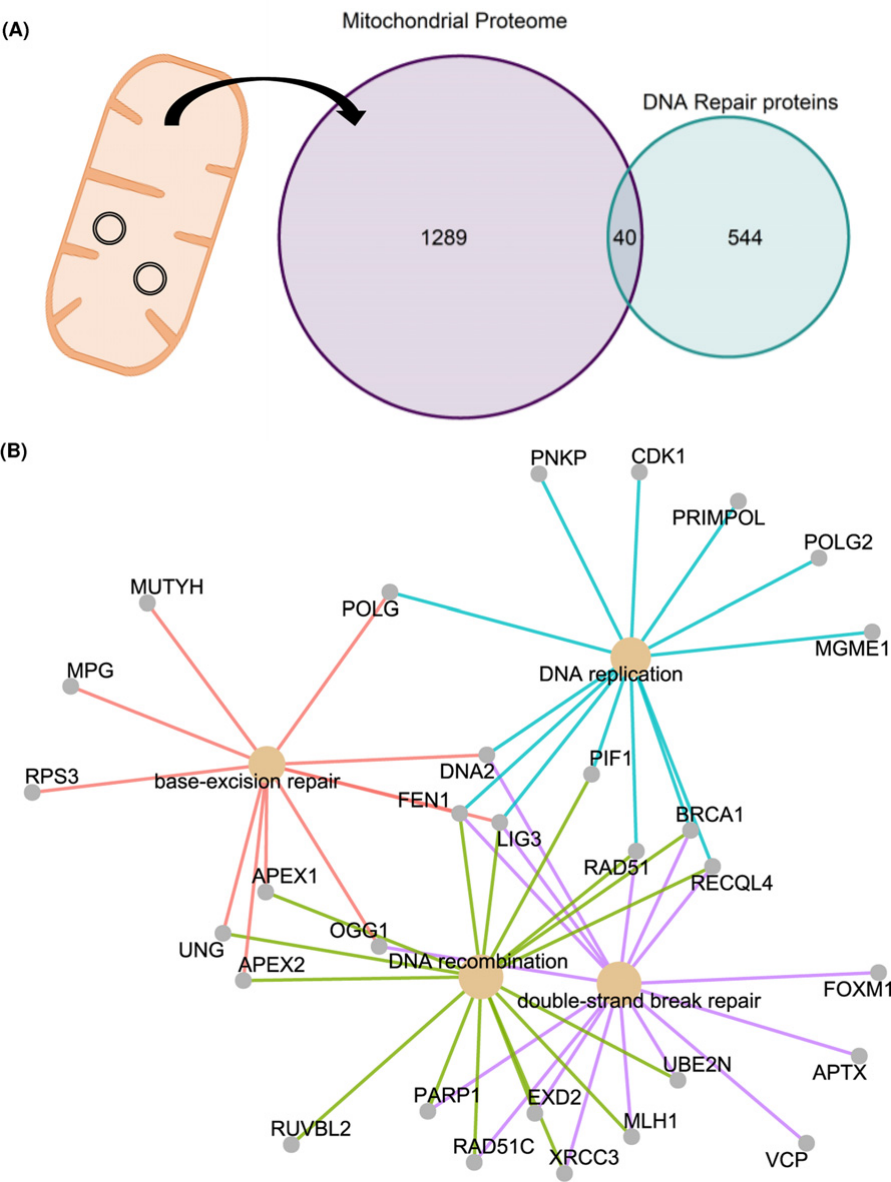
Gene ontology enrichment analysis of DNA repair proteins in the mitochondrial proteome reveals BER and DNA recombination as enriched biological processes (**A**) Protein products of genes annotated with the ‘DNA repair’ gene ontology were screened against the curated IMPI. Forty candidate DNA repair proteins were identified in the mitochondrial proteome representing multiple DNA repair pathways. Black circles represent circular mtDNA in a mitochondrion (orange/pale orange). (**B**) GO enrichment analysis of the 40 candidate genes was performed using clusterProfileR to identify associated biological processes [33]. Analysis reveals BER, DNA recombination, DSB repair and DNA replication as the four most enriched biological processes.

**Figure 2:**
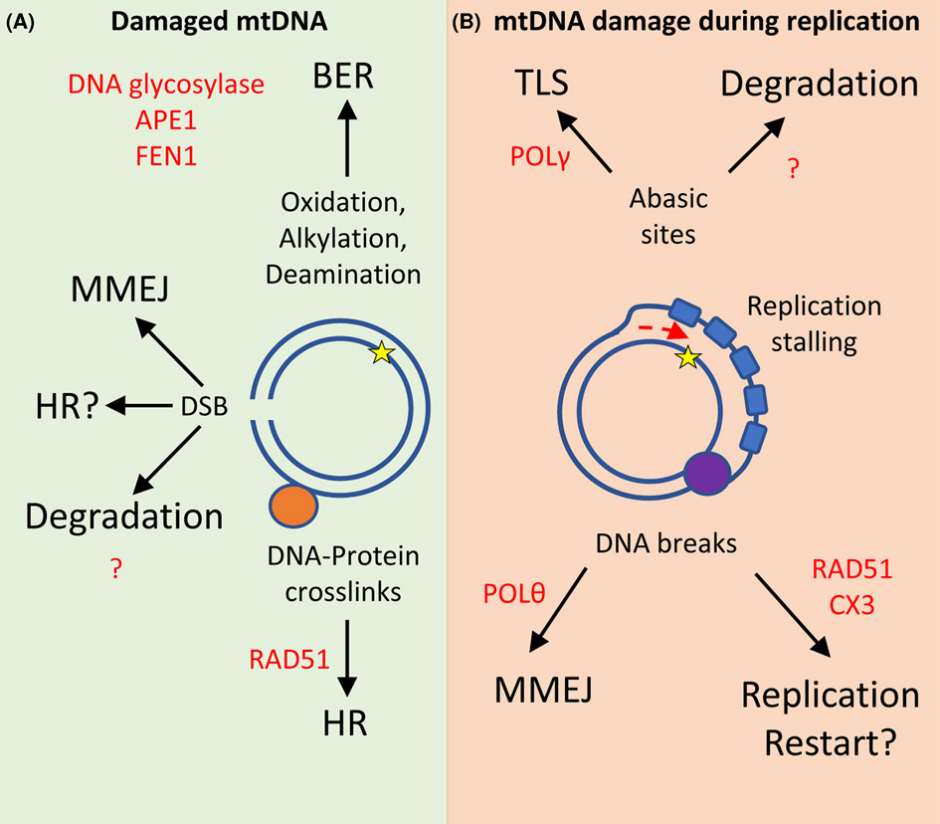
Multiple fates for damaged mtDNA (**A**) The mtDNA (blue circles) encounter several base lesions including oxidation, alkylation and deamination which are primarily repaired by BER. In contrast, DSBs are repaired either by MMEJ or trigger DNA degradation by a yet unknown mechanism. HR repair of protein–DNA cross-links requires both homologous template sequences and RAD51 to repair damage. DSBs may also be processed and repaired by HR. (**B**) During replication, abasic sites generated through BER processing are either repaired by TLS using gap filling activity of POLγ or are targeted for degradation. Lesions present in mtDNA during replication may lead to replication stalling and generation of further DNA breaks which can be repaired by MMEJ or potentially using the non-canonical replication restart functions of RAD51C and XRCC3 (CX3) alongside RAD51. Purple circle represents TWINKLE helicase and Blue rectangles represent mtSSB loading on the displaced strand, red arrow indicates direction of DNA synthesis. Red text indicates key proteins identified in each process. Abbreviations: mtSSB, mitochondrial single-stranded binding protein; TLS, translesion synthesis.

The Integrated Mitochondrial Protein Index (IMPI-2021-Q3pre) has proved to be a powerful resource for probing the mitochondrial proteome – the current iteration of the curated list comprises over 1300 mitochondrially localised proteins [28]. This dataset provides an invaluable snapshot of all the genes with protein products in which strong evidence for mitochondrial localisation has been collated. In the nuclear genome, 584 genes have been annotated with the ‘DNA repair’ Gene Ontology annotation (GO:0006281) [29]. A list of mitochondrially localised DNA repair proteins can be generated by searching for protein products of the DNA repair gene candidates against IMPI (Figure 1A). This approach identifies 40 candidates from the current iteration of IMPI and gene ontologies (full list in Supplementary Table S1). Unsurprisingly, many proteins required for BER are among these candidates including the glycosylases, OGG1, MutY DNA Glycosylase (MUTYH), N-Methylpurine DNA Glycosylase (MPG) and Uracil-DNA Glycosylase (UNG) as well as the flap endonuclease, FEN1 [30,31]. However, many more candidates known to function in other DNA repair pathways are also present such as RAD51 recombinase and the two RAD51 paralogue proteins, RAD51C and XRCC3 [32]. Gene ontology enrichment analysis using the R package, clusterProfileR, reveals that in addition to BER, double-strand break (DSB) repair and DNA recombination gene ontologies are also significantly enriched among the list of candidates (Figure 1B) [33]. Despite this, the mechanistic role of many of these proteins and the DNA repair pathways that can operate within mammalian mitochondria remain unclear. However, depletion or deletion of a number of these proteins does have an impact on mitochondrial genome stability and integrity [34–36]. It is interesting to note that the individual biological processes identified do not necessarily contain the full complement of machinery classically required to process canonical lesions. Potentially, they may use a combination of various pathways or the involvement of non-canonical roles of these proteins that illustrates their importance in mitochondrial genome maintenance. In the following sections, evidence for an expanded repertoire of mtDNA repair pathways is discussed alongside potential interplay between mtDNA repair and replication based on emerging evidence as well as insights from the nuclear genome (overview provided in Figure 2A,B).

## BER in maintenance of genetic integrity

Notwithstanding the overwhelming importance of maintaining the genomic stability of the mitochondrial genome, the mechanisms that protect and repair mtDNA remain largely understudied. For many years, mitochondria were thought to be devoid of the mechanisms necessary to repair DNA lesions supported by the persistence of mtDNA damage beyond that of the nuclear genome [37,38]. Elevated oxidative mtDNA damage likely results from cellular respiration and the generation of reactive oxygen species (ROS) which in turn can modify DNA bases [37]. However, research has shown that mitochondria can repair certain types of damage including abasic sites which are generated and processed by the BER machinery [39,40].

BER enables the repair of damaged bases and requires direct recognition of the lesion by mitochondrially targeted DNA glycosylases [41]. DNA glycosylase enzymes directly bind to and ‘flip-out’ the damaged base from the DNA helix as well as catalysing the removal of the damaged base for further processing [42]. Several mammalian DNA glycosylases recognise an array of DNA base lesions caused by either oxidation, alkylation or deamination [30]. One of the best characterised mitochondrial examples is recognition of 8-oxoguanine by the bifunctional DNA glycosylase, OGG1. Activity of OGG1 on the oxidised base generates a single-strand gap that can be filled by the mitochondrial polymerase, POLγ, and sealed by DNA ligase III [43–45]. DNA glycosylases can either be monofunctional (comprising solely glycosylase activity) which subsequently requires the apurinic/apyrimidinic endonuclease activity of APE1 for further processing, or bifunctional (incorporating AP lyase activity) [42]. Following processing by a monofunctional DNA glycosylase, APE1 cleaves the phosphodiester bond converting the abasic site into a single-strand break which can then be filled by POLγ [44]. In addition to POLγ, POLβ has also been shown to localise to the mitochondria and is important for BER [44,46–48]. Recent research by Baptiste et al*.* showed that POLβ was actually more efficient at filling single-strand DNA gaps generated through BER than POLγ [49].

Two variations of BER are possible depending on whether the DNA polymerase inserts a single nucleotide (short-patch BER) or several nucleotides resulting in the displacement of the downstream strand creating a ‘flap’ structure (long-patch BER). The latter requires additional activity by FEN1 to remove this structure and allow ligation to occur. Both short-patch and long-patch BER have been demonstrated to operate in mammalian mitochondria [50]. Interestingly, research by Baptiste et al*.* also demonstrated that POLβ’s mitochondrial activity was stimulated by TWINKLE helicase, but that this did not appear to rely on helicase activity. It would be prudent to note whether the activity of POLβ versus POLγ in gap filling correlates with preference for either short-patch or long-patch BER. Several additional polymerases have also been identified in mammalian mitochondria including POLθ, POLζ and the primase polymerase, PRIMPOL [51–53]. However, their functional importance is yet to be demonstrated, though it is possible that based on their fidelity and nuclear functions that their mitochondrial function may be skewed towards DNA repair rather than replication.

Localisation of the BER machinery appears to be responsive to ROS generation that in turn generate oxidised bases requiring further processing and repair by BER [54]. In support of the idea that mitochondrial nucleoids are discrete mtDNA processing centres, DNA glycosylases alongside POLγ have been shown to be associated with these inner membrane-associated particles [55]. In 2021, Barchiesi et al*.* showed that APE1 has non-canonical activities in binding to and degrading damaged mitochondrial mRNAs containing abasic sites [56]. In support of this, the authors show that the absence of APE1 led to the accumulation of damaged mRNA transcripts and negatively impacts oxidative phosphorylation. Whether any other BER processing enzymes also facilitate quality control of damaged mRNAs in mitochondria is yet to be established.

In contrast with BER, evidence consistently suggests that nucleotide excision repair (NER) in mammalian mitochondria does not occur due to an inability to remove major NER substrates such as pyrimidine dimers and intrastrand cross-links induced by cisplatin [57–59]. However, two types of NER exist in the nucleus, global genome NER (GG-NER) and transcription-coupled NER (TC-NER). Despite the lack of NER proteins found in mitochondria, the Cockayne Syndrome group B (CSB) protein, involved in TC-NER, has been shown to localise to mitochondria although it appears that CSB in this context promotes mitochondrial BER due to the reduced BER incision activity observed in CSB-deficient cells [60]. XPD is also an NER protein, it localises to mitochondria and appears to play a role in repair of oxidation induced damage as mitochondria depleted of XPD fail to repair oxidised bases compared with wildtype controls [61]. Although XPD and CSB appear to suppress accumulation of oxidative damage, their exact role in mtDNA repair and processing of BER intermediates is yet to be established.

## DSB repair in mammalian mitochondria

The ability of mammalian mitochondria to repair DSBs has remained controversial. In 2017, Moretton et al*.* used an inducible mitochondrially targeted restriction enzyme to selectively induce a DSB in the mtDNA [62]. The authors observed that mtDNA decreased over the period of a day suggesting that a degradation-based quality control mechanism removed damaged mtDNA copies as a main response to DSBs. However, DSB repair by MMEJ has been shown to occur in mitochondria and due to its error-prone nature, is thought to be at least partially responsible for deletions that are frequently observed in mtDNA [63]. Interestingly, the authors also looked for evidence of DSB repair by non-homologous end joining (NHEJ) by incubating mitochondrial extracts with blunt-ended DNA substrates but found no evidence of this repair. Mechanistically, POLθ is known to participate in nuclear MMEJ and is one of the additional polymerases recently shown to localise in mitochondria [51,64]. The disparate observations between DSB repair and degradation could correlate with the extensiveness of the damage and the number of breaks present. For example, the expression of a mitochondrially targeted restriction endonuclease may generate more breaks than the DSB machinery can repair, at which point the balance shifts towards removal of damaged mtDNA copies rather than repair. Additionally, DNA breaks introduced by expression of the PstI, the restriction endonuclease isolated from *Providencia stuartii*, generate ‘sticky’ ends which may be more readily processed by degradation mechanisms rather than ligation due to specificity of the repair machinery. Reciprocally, care needs to be taken to ensure that mitochondrial extracts are devoid of contaminating proteins capable of processing recombinant DNA substrates as well as critically appraising whether plasmid-based reporter constructs are truly representative of the mitochondrial genome. This may prove additionally complex due to the sequestration of the mtDNA in discrete nucleoid structures which in many cases will only contain one copy of the mtDNA per nucleoid and would otherwise lack a template for repair [4]. Furthermore, the impact of mitochondrial DSBs appears to signal and elicit changes beyond the mitochondria. Research by Tigano et al*.* uncovered that induction of DSBs in mitochondria using transcription activator-like effector nucleases (TALENS) triggers a type I interferon response in the nucleus, propagated by the release of mitochondrial RNA into the cytoplasm [65]. Moreover, the authors show that cells lacking mtDNA failed to elicit a robust interferon response following irradiation further indicating the fundamental role for mtDNA to nuclear signalling in this process.

### HR

In the nuclear genome, HR offers a largely error-free mechanism of DNA repair limited to the S- and G_2_-phases of the cell cycle when a homologous template is available to facilitate accurate repair [66]. Due to the presence of many copies of the mitochondrial genome, HR would in theory offer an accurate repair mechanism that could occur independently of the cell cycle. However, the existence of HR in mitochondria has been disputed and due to the error-free nature of repair this would not generate sequence-level changes that could easily be identified or tracked.

In the budding yeast model organism, *Saccharomyces cerevisiae*, recombination of mtDNA has been well-characterised from the initial observation that recombination of genetic material could transfer genes conferring drug resistance to the generation of a mitochondrial recombination hotspot map [67,68]. In mammalian cells however, the existence of DNA recombination in mitochondria has largely been discounted, at least for germline recombination events due to the absence of variation of heteroplasmy over many generations in a mouse model [69]. However, evidence for the capability of mitochondria to perform HR as a means of repairing DSBs, or at least a variation of it, is stacking up.

In the nucleus, HR requires more extensive processing of the DSB to yield a region of single-stranded DNA (ssDNA) via the activity of endo- and exonucleases such as MRE11, CTIP, DNA2 and EXO1 [66]. The ssDNA generated becomes coated in replication protein A (RPA) which prevents the formation of unwanted secondary structures. RPA is then exchanged for the recombinase, RAD51, which forms a nucleoprotein filament on the ssDNA [66]. RAD51 drives strand exchange with a homologous template leading to the displacement of the complementary strand of the template creating a displacement loop (D-loop) [66]. If the second broken DNA end is captured by the displaced loop a double Holliday junction is formed [66]. DNA is then copied through DNA synthesis and branch migration of the Holliday junctions using the complementary DNA sequence. In the final step, these structures are resolved by the concerted activities of endonucleases which yield either a crossover or non-crossover event dependent upon the orientation of the cuts made [70].

Evidence for HR in mammalian mitochondria originated from the use of mitochondrial extracts which are capable of catalysing HR in a plasmid reporter system [71]. Fundamentally, this indicated that the mitochondria contain the machinery required to process DSBs using HR. Additional studies, also using a plasmid reporter system to monitor HR, demonstrated that certain tissue types had a greater propensity for HR including mitochondrial extracts from the testes, brain, kidney and spleen [72]. As with nuclear HR, mitochondrial HR efficiency is attenuated by the immunodepletion of the RAD51, MRE11 or NIBRIN (also known as NBS-1; Nijmegen Breakage Syndrome 1) from mitochondrial extracts prior to incubation with DNA substrates.

In mouse oocytes, loss of RAD51 leads to arrested meiosis during metaphase I accompanied by impaired ATP production, reduced mitochondrial membrane potential and a decrease in mtDNA copy number [73]. The requirement for RAD51 is closely linked to replication of mtDNA as RAD51 is readily localised to mitochondria following replication stress [36]. Several additional proteins required for HR are also found within the mitochondria. The RAD51 paralogues proteins structurally resemble RAD51 but are not capable of compensating for the function of each other, thereby indicating that they each serve unique functions (reviewed by [32]). There are five canonical RAD51 paralogues comprising RAD51B, RAD51C, RAD51D, XRCC2 and XRCC3 which form at least two distinct complexes, the BCDX2 complex and the CX3 complex [74]. Only two RAD51 paralogue proteins are among the mitochondrial proteome, RAD51C and XRCC3. Sage et al*.* showed that depletion of either RAD51, RAD51C or XRCC3 using siRNAs leads to decreased mtDNA copy number instead of the compensatory increase typically observed in response to oxidative stress [35].

Recently Chesner et al*.* suggests that mtDNA–protein cross-links can be repaired using HR in mammalian cells [75]. The authors used a PCR-based assay that delivered cross-linked plasmid DNA into isolated mitochondria. They proposed that DNA repair predominantly occurred by HR on the basis that successful repair was observed when the plasmid contained homology to the mitochondrial genome, whereas no repair was observed in plasmids lacking homology. Furthermore, they observed that no repair occurred when RAD51 polymerisation was inhibited by the small molecule inhibitor, B02 [76]. HR and DNA replication closely cooperate within the nucleus to permit tolerance and bypass of DNA lesions sharing many of the same key proteins including RAD51 and the RAD51 paralogue proteins, however mtDNA replication is functionally distinct from nuclear DNA replication [77,78].

## MtDNA replication

The mitochondrial genome comprises both ‘heavy’ and ‘light’ strands referring to the nucleotide composition of the two strands in relation to their mass [78]. Uniquely, both the heavy and light strands contain their own origins of replication, referred to as O_H_ and O_L_ respectively. The O_L_ replication origin sits approximately two-thirds of the way round the mtDNA molecule from the O_H_ locus. As part of the strand-displacement model, mtDNA replication initiates at O_H_ with the aid of the mitochondrial RNA polymerase (POLRMT) which synthesises an RNA primer to enable extended DNA synthesis by POLγ [79]. Replication then proceeds unidirectionally around the mitochondrial genome to synthesise the new H-strand [78]. TWINKLE helicase unwinds and displaces the complementary L-strand during this process to create a D-loop which becomes coated in the mitochondrial single-stranded binding protein (mtSSB) [80,81]. When the helicase reaches the O_L_ locus, displacement of the light strand results in the formation of a stem–loop structure in this region which prevents the binding of mtSSB [82]. POLRMT is then able to initiate synthesis of the reverse L-strand by incorporating an RNA primer that can then be used again by POLγ in the reverse direction [83]. This synthesis continues until both strands have been fully replicated. A variation of this model referred to as RITOLS proposes that the lagging strand replication occurs concurrently with that of the leading strand but RNA in incorporated rather than DNA (reviewed in [84]). Following DNA synthesis, RNA is then removed by RNAse H1 and the DNA ends are ligated by DNA ligase III [84]. In the final step of mtDNA replication, the two daughter copies must be separated. Relieving of the topological stress induced by replication is catalysed primarily by the mitochondrial topoisomerase, TOP1MT [85].

### Replication-associated repair

An emerging picture is developing in understanding the intricate interplay between the distinct repair pathways and DNA replication. Processing of modified bases by the BER machinery generates abasic or ssDNA breaks depending on the stage of processing or whether the glycosylase is mono- or bifunctional [42]. In the nucleus, if a progressing replication fork encounters one of these intermediates, it will lead to stalling of the fork [86]. To maintain the stability and bypass this structure, the cell can either remodel the replication fork by annealing the two nascent strands to one another allowing synthesis of complementary sequences past the lesion followed by a strand (reviewed in [77]). This creates a ‘chicken-foot’ structure that is stabilised by RAD51 and the BCDX2 complex [87]. To continue replicating the DNA this structure is degraded by nucleases to yield a section of ssDNA that can strand invade ahead of the lesion to permit synthesis [88]. The complementary strand can then be synthesised in the reverse direction using this new template to continue DNA replication [77]. Alternatively, a second recombination-dependent mechanism can permit the continuation of DNA replication – this requires the controlled collapse of the replication fork to generate a single-ended DSB [89]. The single-ended DSB is then processed to reveal a region of ssDNA that can then drive strand invasion to restart the fork. If the DNA lesion has already been processed to generate a single-strand break this would be converted into a DSB upon replication [90]. In which case, fork restart would potentially be favoured in response to ssDNA BER intermediates. Interestingly, only RAD51C and XRCC3 along with RAD51 are required to perform the latter recombination-based restart in the nucleus, with the CX3 complex having a functionally distinct role from that of the BCDX2 complex [87,91]. Furthermore, RAD51C was recently shown to cooperate with ALKBH3 to facilitate lesion recognition and bypass of alkylation-induced damage (N3-methylcytosine residues), which would otherwise lead to fork stalling and genomic instability [92]. In mitochondria, RAD51, RAD51C and XRCC3 have been shown to localise at D-loops and are enriched in mitochondrial nucleoids following replication stress caused by dideoxycytidine, which stalls mitochondrial replication specifically [34]. In addition, recruitment of RAD51, RAD51C and XRCC3 is dependent upon TWINKLE helicase indicating close cooperation of these factors with replication. Interestingly, POLγ enrichment on mtDNA also appears to be maintained by the presence of RAD51C and XRCC3. Recently, Zhao et al. (2021) identified XRCC2 as a potential prognostic factor for hepatocellular carcinoma [93]. The authors showed that XRCC2 was found in the mitochondrial fraction of cell extracts and that its depletion led to an accumulation of mtDNA damage. This raises further questions as to whether the DNA protection function of XRCC2 in mitochondria requires the formation of a complex or reflects the more recently discovered non-canonical function of XRCC2 in sensing dNTP pool fluctuations [94]. Furthermore, the exact role of these proteins in mitochondria remains to be established, however the non-canonical roles of the proteins in the nuclear DNA replication may potentially provide useful insights for further study.

If BER intermediates persist, the question remains as to how efficiently abasic sites are repaired within mammalian mitochondria – this has been challenging to study as murine APE1 knockouts, that would lead to accumulation of abasic sites, are embryonic lethal [95]. Authors overcame this limitation via generation of a haploinsufficient APE1 (+/−) transgenic mouse which displayed decreased mtDNA abundance suggesting that mtDNA containing abasic sites was being degraded [95]. However, abasic lesions can also be bypassed by translesion synthesis (TLS) using POLγ, though evidence suggest the efficiency of this is quite low and quickly shifts in favour of mtDNA degradation rather than lesion bypass when the number of abasic sites increases [96].

Fanconi anaemia (FA) is a rare heritable disorder characterised by developmental defects, bone marrow failure and an increased risk of haematological malignancies [97]. Characteristically, FA patient cells display increased genetic instability resulting from mutations in genes governing the stability and protection of DNA replication forks from interstrand cross-links [98]. These genes found to be mutated in FA are prefaced with the ‘FANC’ designation followed by alphanumeric characters denoting the complementation group. Processing of interstrand cross-links requires the controlled collapse of replication forks by the ‘unhooking’ of one of the strands to be replicated thereby generating a single-ended DSB [99]. This process is mediated by the FANCD2 and FANCI heterodimer which becomes monoubiquitinated prior to recognition of the lesion [100]. Monoubiquitination of FANCD2–FANCI is facilitated by a core complex comprising at least eight proteins [99]. Once the cross-linked base is unhooked, TLS allows synthesis past the lesion which can then be removed by NER [99]. In the final stage of the repair process the single-ended DSB strand invades the repaired DNA to restart the replication fork [99]. Two genes acting downstream of FA core complex processing have already been discussed are associated with FA complementation groups, FANCR^RAD51^ and FANCO^RAD51C^ [101,102]. Several additional genes with protein products required for HR are also involved in this process including FANCD1^BRCA2^, FANCS^BRCA1^, FANCJ^BRIP1^, FANCN^PALB2^ [103–106].

In support of their mitochondrial role, research has revealed that cells taken from FA patients or cells with mutations in genes associated with some of the complementation groups display mitochondrial dysfunction and increased generation of ROS [107,108]. More recently, FA proteins have been associated with the mitochondrial stress linked to instability of common fragile sites and appear to be involved in mitophagy [109,110]. The exact function of these proteins in protecting mitochondria remains largely enigmatic, however recent research by Luzwick et al*.* showed that protection of mitochondrial forks required several proteins including MRE11, FANCD2, RAD51C, BRCA2 and the endonuclease, SLX4, in response to stalling of mitochondrial replication with dideoxycytidine [111]. Interestingly, the authors noted that neither FANCD2 monoubiquitination nor members of the FA ‘core’ complex are required for the mitochondrial fork protection activity observed when assaying nascent mtDNA synthesis. However, some have reported that at least FANCD2 (with or without ubiquitination) appears to be important for mitochondrial function [109,112,113]. Critically, the involvement of some common factors, but not others to elicit a robust response suggest interplay between existing mechanisms.

MtDNA replication is also the source of an mtDNA deletion associated with Kearns–Sayre syndrome [114]. The ‘common deletion’ which is attributed to loss of a 4977-bp segment of the mitochondrial genome is associated with pigmentary retinopathy, cerebellar ataxia and neuromuscular defects including weakness of the eye muscles (Opthalmoplegia) [115,116]. By developing a novel technique to temporally dual label mtDNA during replication, Phillips et al*.* demonstrated that DSBs induced during replication were responsible for the deletion by attempting to repair the breaks by error-prone MMEJ [114]. Furthermore, the authors approach provided strong evidence for the strand-displacement model of mtDNA replication, as two distinct modes of labelling were observed (representing the firing of the O_H_ and O_L_ independently of one another).

After mtDNA replication, DNA mismatches introduced by TLS can be corrected using mismatch repair (MMR), which unlike nuclear MMR requires YB-1 instead of heterodimers comprising MLH and MSH proteins [117,118]. Table 1 summarises the evidence discussed for an expanded repertoire of DNA repair mechanisms in mammalian mitochondria alongside highlighting key proteins involved in each stage of repair. Critically, the full complement of factors required to elicit most of these repair process in mitochondria have yet to be identified. Ultimately further research will be required to tease out these specific mechanisms and determine their similarity or difference with existing mechanisms characterised in the repair of nuclear DNA damage.

**Table 1:** Evidence for mtDNA repair pathways discussed and key proteins involved in each stage of repair

DNA repair pathway	Stages of repair	Key mitochondrial proteins	References
BER	Base excision and 3′ OH generation	OGG1, MUTYH, MPG, UNG1, NTH1, NIEL1, NIEL2	[23,26,27,30,37,119–121]
	DNA synthesis	POLγ, POLβ	[44,48]
	Flap removal and ligation	FEN1, LIG3	[31,45]
HR	DNA end resection and strand exchange	RAD51, RAD51C, XRCC2, XRCC3	[34,72,75]
	Resolution	?	
MMEJ	Base pairing, DNA synthesis and ligation	POLθ, LIG3	[63,64,114]
Replication-associated repair	Fork protection	MRE11, XRCC2	[93,111]
	Fork restart	RAD51, RAD51C, XRCC3	[34,36,111]
TLS	Lesion bypass	POLγ	[96]
MMR	Lesion recognition and replacement	YB-1	[117]

## Summary

In summary, significant strides have been made to identify and characterise the mechanisms that protect the integrity of the mitochondrial genome. This expanded portfolio of mechanisms comprising at least BER, MMEJ, TLS, MMR with emerging evidence for HR and HR mediated restart of stalled mitochondrial replication [41,42,63,96,117]. Critically, this suggests that mammalian mitochondria are better equipped than previously thought to tolerate and repair the high levels of damaging agents they are exposed to through cellular respiration [37]. Despite many known nuclear DNA repair proteins being shown to localise in mitochondria, the functional importance of these proteins in the context of DNA repair remains enigmatic. Nuclear functions of these proteins have provided important insights into their likely mitochondrial role; however, the development of novel assays to assess mtDNA repair and replication may yield important insights in future to help characterise these mechanisms. Critically, unlike the well-characterised mitochondrial BER pathway, other repair mechanisms do not necessarily have a full complement of proteins to enable mtDNA repair to occur like-for-like with the nuclear DNA. As a result, it remains a possibility that combinations of repair pathways offer mechanisms to tolerate DNA lesions, which potentially may be coupled with mtDNA replication to permit a diverse response to differing DNA lesions. Indeed, further identification of non-canonical roles of mitochondrially localised proteins may also offer important insights, whether closely aligned to DNA repair and replication or unique functions such as recognition and degradation of damaged mRNAs [56]. Proving the existence and feasibility of repair using these mechanisms relies on accurate evaluation and validation of the cellular compartmentalisation of these proteins. Proteomic evidence has provided a significant snapshot into mitochondrial localisation, however greater questions remain in establishing how mitochondrial localisation is achieved, particularly when mitochondrial localisation appears responsive to damage [54]. Challenges also remain on whether localisation can truly accurately be predicted on genetic, transcript or peptide sequences alone or whether the protein–protein interactome can also play a significant role. Lastly, discovery of the mechanism governing the degradation of mtDNA will be critical to understanding the potential interplay and balancing act between repair and degradation [62].

## Supplementary Material

Supplementary Table S1

## Data Availability

Data will be made available on request.

## Abbreviations

APE1: apurinic/apyrimidinic endonuclease I
BCDX2: RAD51B-RAD51C-RAD51D-XRCC2 heterotetrameric complex
BER: base excision repair
CSB: Cockayne Syndrome group B
CX3: RAD51C-XRCC3 heterodimeric complex
dNTP: deoxynucleoside triphosphate
DSB: double-strand break
D-loop: displacement loop
FA: Fanconi anaemia
FEN1: flap endonuclease I
HR: homologous recombination
IMPI: Integrated Mitochondrial Protein Index
MDDS: mitochondrial DNA depletion syndrome
MMEJ: microhomology-mediated end joining
MMR: mismatch repair
MPG: N-methylpurine DNA glycosylase
mtDNA: mitochondrial DNA
mtSSB: mitochondrial single-stranded binding protein
NER: nucleotide excision repair
NHEJ: non-homologous end joining
OGG1: 8-oxoguanine DNA glycosylase
POLγ: polymerase γ
ROS: reactive oxygen species
RPA: replication protein A
ssDNA: single-stranded DNA
TC-NER: transcription-coupled NER
TLS: translesion synthesis
UNG: uracil-DNA glycosylase
