# Supplementary material for: Beyond base excision repair: an evolving picture of mitochondrial DNA repair
Source: Biosci Rep. 2021 Oct 14;41(10):BSR20211320. doi: 10.1042/BSR20211320 (PMC8527207; doi:10.1042/BSR20211320)
Supplement: Supplementary Table S1 [file BSR-2021-1320C_supp.pdf]

Supplemental table 1

No.	Ensembl Gene ID	Gene Symbol	Description
1	ENSG00000125871	MGME1	mitochondrial genome maintenance exonuclease 1
2	ENSG00000004142	POLDIP2	polymerase (DNA-directed), delta interacting protein 2
3	ENSG00000091483	FH	fumarate hydratase
4	ENSG00000140521	POLG	polymerase (DNA directed), gamma
5	ENSG00000256525	POLG2	polymerase (DNA directed), gamma 2, accessory subunit
6	ENSG00000149273	RPS3	ribosomal protein S3
7	ENSG00000081177	EXD2	exonuclease 3-5 domain containing 2
8	ENSG00000138346	DNA2	DNA replication helicase/nuclease 2
9	ENSG00000014824	SLC30A9	solute carrier family 30 (zinc transporter), member 9
10	ENSG00000116288	PARK7	parkinson protein 7
11	ENSG00000169188	APEX2	APEX nuclease (apurinic/apyrimidinic endonuclease) 2
12	ENSG00000100823	APEX1	APEX nuclease (multifunctional DNA repair enzyme) 1
13	ENSG00000114026	OGG1	8-oxoguanine DNA glycosylase
14	ENSG00000132781	MUTYH	mutY DNA glycosylase
15	ENSG00000177889	UBE2N	ubiquitin-conjugating enzyme E2N
16	ENSG00000076248	UNG	uracil DNA glycosylase
17	ENSG00000143799	PARP1	poly (ADP-ribose) polymerase 1
18	ENSG00000100601	ALKBH1	alkB homolog 1, histone H2A dioxygenase
19	ENSG00000168496	FEN1	flap structure-specific endonuclease 1
20	ENSG00000051180	RAD51	RAD51 recombinase
21	ENSG00000106268	NUDT1	nudix (nucleoside diphosphate linked moiety X)-type motif 1
22	ENSG00000078237	TIGAR	TP53 induced glycolysis regulatory phosphatase
23	ENSG00000103152	MPG	N-methylpurine DNA glycosylase
24	ENSG00000108384	RAD51C	RAD51 paralog C
25	ENSG00000221829	FANCG	Fanconi anemia, complementation group G
26	ENSG00000005156	LIG3	ligase III, DNA, ATP-dependent
27	ENSG00000165280	VCP	valosin containing protein
28	ENSG00000160957	RECQL4	RecQ protein-like 4
29	ENSG00000126215	XRCC3	X-ray repair complementing defective repair in Chinese hamster cells 3
30	ENSG00000182150	ERCC6L2	excision repair cross-complementation group 6-like 2
31	ENSG00000164306	PRIMPOL	primase and polymerase (DNA-directed)
32	ENSG00000183207	RUVBL2	RuvB-like AAA ATPase 2
33	ENSG00000012048	BRCA1	breast cancer 1, early onset
34	ENSG00000049167	ERCC8	excision repair cross-complementation group 8
35	ENSG00000170312	CDK1	cyclin-dependent kinase 1
36	ENSG00000111206	FOXM1	forkhead box M1
37	ENSG00000140451	PIF1	PIF1 5-to-3 DNA helicase
38	ENSG00000039650	PNKP	polynucleotide kinase 3-phosphatase
39	ENSG00000137074	APTX	aprataxin
40	ENSG00000076242	MLH1	mutL homolog 1

Full list of DNA repair GO-annotated genes with protein products found in the integrated mitochondrial protein index.
